# Transthyretin deposition alters cardiomyocyte sarcomeric architecture, calcium transients, and contractile force

**DOI:** 10.14814/phy2.15207

**Published:** 2022-03-09

**Authors:** Kyle T. Dittloff, Emanuele Spanghero, Christopher Solís, Kathrin Banach, Brenda Russell

**Affiliations:** ^1^ Department of Physiology and Biophysics University of Illinois at Chicago Chicago Illinois USA; ^2^ Department of Biomedical Engineering University of Illinois at Chicago Chicago Illinois USA; ^3^ Department of Internal Medicine/Cardiology Rush University Medical Center Chicago Illinois USA

**Keywords:** aging, amyloidosis, electromechanical uncoupling, heart failure, HFpEF, transthyretin

## Abstract

Age‐related wild‐type transthyretin amyloidosis (wtATTR) is characterized by systemic deposition of amyloidogenic fibrils of misfolded transthyretin (TTR) in the connective tissue of many organs. In the heart, this leads to age‐related heart failure with preserved ejection fraction (HFpEF). The hypothesis tested is that TTR deposited in vitro disrupts cardiac myocyte cell‐to‐cell and cell‐to‐matrix adhesion complexes, resulting in altered calcium handling, force generation, and sarcomeric disorganization. Human iPSC‐derived cardiomyocytes and neonatal rat ventricular myocytes (NRVMs), when grown on TTR‐coated polymeric substrata mimicking the stiffness of the healthy human myocardium (10 kPa), had decreased contraction and relaxation velocities as well as decreased force production measured using traction force microscopy. Both NRVMs and adult mouse atrial cardiomyocytes had altered calcium kinetics with prolonged transients when cultured on TTR fibril‐coated substrates. Furthermore, NRVMs grown on stiff (~GPa), flat or microgrooved substrates coated with TTR fibrils exhibited significantly decreased intercellular electrical coupling as shown by FRAP dynamics of cells loaded with the gap junction‐permeable dye calcein‐AM, along with decreased gap junction content as determined by quantitative connexin 43 staining. Significant sarcomeric disorganization and loss of sarcomere content, with increased ubiquitin localization to the sarcomere, were seen in NRVMs on various TTR fibril‐coated substrata. TTR presence decreased intercellular mechanical junctions as evidenced by quantitative immunofluorescence staining of N‐cadherin and vinculin. Current therapies for wtATTR are cost‐prohibitive and only slow the disease progression; therefore, better understanding of cardiomyocyte maladaptation induced by TTR amyloid may identify novel therapeutic targets.

## Graphical abstract

Transthyretin (TTR) fibril deposition in the heart is a common cause of cardiac dysfunction and failure with preserved ejection fraction (HFpEF) in the elderly. Studies show that TTR fibrils alter primary cardiac myocyte function with decreased contraction and relaxation velocities, reduced force production, prolonged calcium transients, and decreased intercellular electrical coupling. Structurally, the TTR caused sarcomere disorganization, reduced electromechanical junctions, and increased sarcomeric ubiquitin localization. These findings may suggest new therapeutic approaches for cardiac amyloidosis.

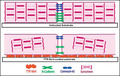

## 1 INTRODUCTION

Heart failure is a significant health concern that affects an estimated 2.1% of American adults, with the prevalence expected to rise to 3% by 2030 (Virani et al., 2021). There are numerous underlying causes for heart disease, but among the least studied is cardiac amyloidosis, which is caused by the deposition of misfolded protein into the cardiac extracellular matrix (ECM). One common form of cardiac amyloidosis is due to deposition of insoluble, misfolded transthyretin (TTR) fibrils due to rare genetic point mutations, and more commonly from aging (Kittleson et al., 2020). Normal TTR exists as a 55 kDa homotetramer that transports thyroxine and retinol‐binding protein in the bloodstream. Point mutations and aging can cause the tetramer to become thermodynamically unstable, leading to dissociation into monomeric species. These monomers self‐aggregate into oligomeric and eventual fibrillar species in various tissues such as the peripheral nerves and the heart (Saelices et al., 2015). Deposition of misfolded, wild‐type TTR in aging, known as wtATTR, is now diagnosed by non‐invasive clinical advances (Witteles et al., 2019). It is estimated that in over 10% of elderly patients, heart failure with preserved ejection fraction (HFpEF) is caused by wtATTR (González‐López et al., 2015).

Clinically, wtATTR manifests with a wide range of cardiac symptoms including increased ventricular stiffness and wall thickness, with frequent occurrence of atrial fibrillation and arrhythmia due to electromechanical uncoupling caused by the amyloid fibril infiltration (Siddiqi & Ruberg, 2018). Medical therapies can treat symptomatic arrhythmias, but cardiomyopathy resulting from wtATTR progresses to heart failure. However, standard heart failure therapies often provide no clinical benefit to these patients with the only recourse being a heart transplant (Kittleson et al., 2020).

Currently, the sole FDA‐approved therapy for wtATTR is tafamidis, which limits disease progression by stabilizing the TTR tetramer to prevent unfolding and subsequent amyloid fibril formation (Shah, 2019). However, this is currently the most expensive FDA‐approved cardiovascular therapy to date and thus inaccessible to many patients Kazi et al. (2020). Nonetheless, a better basic understanding of amyloidogenic TTR effects on cardiomyocytes is still needed in the hopes of innovative therapies.

Transgenic rodent models wtATTR generally do not produce cardiac deposition of TTR fibrils, necessitating alternative in vitro approaches (Ibrahim et al., 2019). Short‐term in vivo rat models in which a bolus of aggregated TTR is injected into the heart apex have been shown to recapitulate some aspects of wtATTR (George et al., 2020). In vitro, it has been demonstrated that isolated cardiomyocytes, when cultured with various TTR species in the culture media, exhibit cytotoxicity with increased ROS production and altered calcium transient and action potential repolarization profiles (Sartiani et al., 2016). Additionally, human‐induced pluripotent stem cell‐derived cardiomyocytes (iPSC‐CM) cultured with plasma from wtATTR exhibit impaired growth responses compared to iPSC‐CM cultured with plasma from healthy patients (Hein, Furkel, et al., 2021). Here, the focus is to understand how underlying TTR fibrils affect cardiomyocytes cultured on different engineered substrata in order to best recapitulate cardiomyocyte biology in vitro. This study reports how the deposition of TTR affects cardiomyocyte calcium transients, contractility parameters and force production, mechanical and electrical junction structure and function, and sarcomere architecture.

## 2 METHODS

### 2.1 TTR fibril production

Recombinant human transthyretin (TTR) protein (Invitrogen cat# LF‐P0054) was induced to a fibrillar state according to previously described protocols (Groenning et al., 2015). Briefly, 1‐M acetic acid and 2‐M NaCl were added to TTR protein reconstituted in water at 0.5 mg/ml, to a final concentration of 50‐mM acetic acid and 100‐mM NaCl (pH 3.0). Fibril self‐assembly was allowed to occur for at least 72 h and fibrils were stored at 4°C for subsequent use in experiments. As previously shown, commercially available recombinant human TTR is over 95% pure via SDS‐PAGE, possible lipopolysaccharide (LPS) contamination resulting from the *E. coli* expression system was tested with the Pierce Endotoxin Quant Kit (Thermo Fisher cat# A39553) and found to be in the range of commercially available “low endotoxin” TTR (Dittloff et al., 2021).

### 2.2 TTR fibril deposition on substrata with variable stiffness or microgroove topography

Glass or polystyrene culture dishes were coated with 10‐µg/ml fibronectin with or without the addition of 50‐µg/ml TTR fibrils or native tetramer in phosphate‐buffered saline (PBS) solution. Dishes were coated for 2 h in a 37°C, 5% CO_2_ incubator before cell plating.

Microgrooved substrates were prepared by molding 400 kPa polydimethylsiloxane (PDMS) from a parylene template to produce spaced grooves as done previously, with grooves 10‐µm wide, 5‐µm high, and ridges 10‐µm wide (Motlagh, Senyo, et al., 2003). Before coating with fibronectin or TTR fibrils, PDMS microgroove substrates were functionalized with 3‐aminopropyl triethoxysilane (Sigma‐Aldrich cat# 440140).

Polyacrylamide (PAA) substrates with a stiffness of 10 kPa were prepared as previously described (Li et al., 2016). Before polymerization, fluorescent microspheres (Invitrogen cat# F8807) were included in substrates for later traction force microscopy (Broughton et al., 2016; Ribeiro et al., 2017). PAA substrates were functionalized with Sulfo‐Sanpah (Thermo Fisher cat# 22589) before coating with fibronectin or TTR fibrils.

### 2.3 Rat neonatal ventricular myocyte and human iPSC‐derived cardiomyocyte culture on various substrata with TTR fibril deposition

Neonatal rat ventricular myocyte (NRVM) isolation: All research animals were obtained and used in accordance with the guidelines of the NIH (National Research Council (US) Institute for Laboratory Animal Research, 1996). Animal studies were approved by the UIC Institutional Animal Care and Use Committee and conducted according to the NIH Guide for the Care and Use of Laboratory Animals. The hearts were removed and cells isolated from 1‐ to 2‐day‐old male and female Sprague–Dawley rats using collagenase type II as previously described (Boateng et al., 2003). Cells were plated in 10‐cm tissue culture dishes and fibroblasts were given 1 h to attach, after which surrounding unattached myocytes were removed and plated onto substrates, and NRVMs were cultured on substrates for 72 h before use.

Human‐induced pluripotent stem cell‐derived cardiomyocyte (iPSC‐CM) culture: Human iPSC‐CM from a male donor was purchased from Fujifilm Cellular Dynamics Intl. (iCell Cardiomyocytes, cat# R1105). Upon thaw, cells were plated on fibronectin or TTR‐coated substrates and maintained for 7 days before assay.

### 2.4 Adult atrial myocyte isolation

Atrial myocytes were isolated from 3‐ to 6‐month‐old male WT (C57/BL6) mice (The Jackson Laboratory). The isolation was performed as previously described (DeSantiago et al., 2013, 2018; Varma et al., 2021). After Langendorff perfusion, the left and right atria were dissected cut into strips and further incubated in digestion buffer (mg/L): 0.1 Liberase TM (Roche), 0.14 trypsin (Gibco/Invitrogen), and 1 Protease type XIV (Sigma; 20 min at 37°C). Cell dissociation was enhanced by gentle cell dispersion with a Pasteur pipette. The digestion was stopped by the addition of bovine calf serum (Hyclone) before step‐wise reintroduction of calcium into the solution. Isolated cells were plated on laminin (1 mg/ml, Sigma‐Aldrich) coated glass coverslips with or without TTR fibrils (2 mg/mL) in standard Tyrode's solution (in mmol/L: NaCl 130, KCl 5.4, CaCl_2_ 1, MgCl_2_ 1.5, Glucose 10, HEPES 5; pH 7.4). Animal procedures were performed with the approval of the IACUC of Rush University and in accordance with the National Institute of Health Guide for the Care and Use of Laboratory Animals.

### 2.5 Calcium transient analysis

NRVMs grown at 80 k cells/cm^2^ on 10 kPa PAA substrates with or without TTR fibrils for 72 h were loaded with 1‐μM Fluo‐4/AM (Thermo Fisher, F14201) in serum‐free maintenance medium for 30 min at 37°C. Dishes were then rinsed with sterile HEPES, and maintenance medium was added for another 30 min at 37°C to allow full de‐esterification of intracellular AM. Video recordings were captured utilizing Fast Airyscan acquisition with a Zeiss LSM880 confocal microscope equipped with temperature and CO_2_ environmental controls. Calcium transient parameters were extracted with the use of a custom MATLAB program. Calcium transients that included a spontaneous release event (characterized by lower amplitude and random occurrence) were not included in the calculation of time to 50%/90% of peak and baseline or *tau* for NRVMs.

Isolated mouse atrial myocytes were loaded (20 min) with the membrane permeable ratiometric dye indo‐1/AM (5 μM). After de‐esterification (20 min), cells were excited at 360 nm and using photomultiplier tubes. Emission was collected at 410 nm (F410) and 485 nm (F485). Fluorescence signals were background subtracted and [Ca^2+^]_i_ changes expressed as changes in the fluorescent ratio (*R* = *F*_410_/*F*_485_) (Florea & Blatter, 2010; Varma et al., 2021). Throughout the experiment, calcium transients were induced by field stimulation (0.5 Hz).

### 2.6 Traction force microscopy

NRVMs were grown at 80 k cells/cm^2^ or iPSC‐CM at 150 k cells/cm^2^ on 10 kPa PAA substrates with or without TTR fibrils for 72 h or 7 days, respectively. Video recordings of contracting single cells were captured utilizing Fast Airyscan acquisition with a Zeiss LSM880 confocal microscope equipped with temperature and CO_2_ environmental controls. In order to calculate contractile and relaxation velocities, as well as force production, displacement of beads embedded in the hydrogel was measured using a published MATLAB program (Ribeiro et al., 2017).

### 2.7 Gap‐FRAP assay

The Gap‐FRAP assay was used to assess gap junction function (Abbaci et al., 2007; Fahrenbach et al., 2007). Briefly, NRVMs were grown at 290 k cells/cm^2^ on microgrooved substrates with or without deposited tetrameric of fibrillar TTR and loaded with 2‐μM gap junction‐permeable calcein‐AM (Thermo Fisher cat# 65‐0853‐39) for 30 min at 37°C, before rinsing with sterile HEPES and returning to the culture medium. Imaging used a Zeiss LSM880 confocal microscope equipped with temperature and CO_2_ environmental controls. An aligned single cell was outlined as a region of interest (ROI) and photobleached. Recovery after photobleaching was monitored for 5 min and quantified relative to the fluorescence of a non‐bleached cell for reference. The rate of recovery and amplitude‐weighted kinetic constant (k_FRAP_) was determined as previously described (Solís & Russell, 2019).

### 2.8 Connexin 43 quantification

NRVMs grown for 72 h on glass fibronectin or fibronectin with TTR fibril substrates were fixed in 10% formalin after 72 h of culture. Cells were permeabilized with 0.1% Triton X‐100 (Sigma‐Aldrich) and probed with a 1:100 antibody for connexin 43 (Cell Signaling Technologies cat#3512) and 1:500 antibody for α‐actinin (Abcam cat#9465) in 1% BSA, 0.1% Tween‐20 solution. Cells were counterstained with secondary antibody (Alexa Fluor 488/ Alexa Fluor 555, respectively, Thermo Fisher) and DAPI‐containing mounting medium (Vector Laboratories) and imaged on a Zeiss LSM880 confocal microscope. A custom CellProfiler (Carpenter et al., 2006) pipeline was used to identify connexin 43 normalized for myocyte nuclei in each field of view. At least 35 images pooled across seven independent biological experiments were used to calculate connexin 43 content.

### 2.9 Sarcomere organization, N‐cadherin quantification

NRVMs grown for 72 h at 290 k cells/cm^2^ on microgrooved substrates with or without deposited TTR were fixed in 10% formalin after 72 h of culture. Cells were permeabilized with 0.1% Triton X‐100 (Sigma‐Aldrich) and probed with a 1:200 antibody for N‐cadherin (Proteintech cat#220818‐1‐AP) and 1:500 antibody for α‐actinin (Abcam cat#9465) in 1% BSA, 0.1% Tween‐20 solution. Cells were counterstained with secondary antibody (Alexa Fluor 488/ Alexa Fluor 555, respectively, Thermo Fisher) and DAPI‐containing mounting medium (Vector Laboratories) and imaged on a Zeiss LSM880 confocal microscope.

To measure sarcomere organization, the Scanning Gradient Fourier Transform (SGFT) method was used (Salick et al., 2020). To measure N‐cadherin content at the intercellular surfaces, a custom CellProfiler pipeline was used to quantify the area occupied by N‐cadherin normalized to the area occupied by α‐actinin in each field of view. At least 15 images pooled across three independent biological experiments were used to calculate N‐cadherin content and sarcomere organization. To quantify N‐cadherin intercellular junctions at the ends of aligned myocytes, intensity profiles of lines drawn parallel to the groove axis were extracted using Fiji, with peaks above baseline counted as an N‐cadherin positive cell junction. The number of junctions was normalized to testing line length in millimeters. At least 15 images pooled across three independent biological experiments were used to quantify the N‐cadherin junctions.

### 2.10 Analysis of sarcomere content

NRVMs grown for 72 h on glass fibronectin or fibronectin with TTR fibril substrates were fixed with rapid methanol fixation at −80°C for 10 min. Sarcomere content was quantified from immunofluorescence images of cardiomyocytes stained with sarcomeric α‐actinin antibodies (Abcam #9465) using global thresholding to classify pixels belonging to Z‐discs or non‐sarcomeres (Otsu, 1979). Sarcomere content was estimated as the sum of the sarcomeric α‐actinin grayscale intensity per pixel divided by the cell area (*I*_sarc_/Area).

### 2.11 Ubiquitin distribution

NRVMs grown for 72 h on glass fibronectin or fibronectin with TTR fibril substrates were fixed with rapid methanol fixation at −80°C for 10 min to prevent extraction of ubiquitin during processing. Ubiquitin content was quantified from immunofluorescence images of cardiomyocytes stained with linkage‐specific oligo‐K48 ubiquitin (Abcam #140601) and sarcomeric α‐actinin antibodies (Abcam #9465). The sarcomeric oligo‐K48 ubiquitin was quantified from regions spanning 0.65 μm away from the α‐actinin‐positive pixels of the Z‐discs. This distance was selected to include most of the I‐ and A‐bands while accounting for shorter sarcomere lengths. Ubiquitin content per cardiomyocyte was quantified as the ratio of sarcomeric oligo‐K48 ubiquitin grayscale intensity per pixel divided by the non‐sarcomeric ubiquitin grayscale intensity excluded per pixel (Ub_sarc_:Ub_non‐sarc_).

### 2.12 Vinculin quantification

NRVMs grown for 72 h on glass fibronectin or fibronectin with TTR fibril substrates were fixed in 10% formalin after 72 h of culture. Cells were permeabilized with 0.1% Triton X‐100 (Sigma‐Aldrich) and probed with a 1:250 antibody for vinculin (Abcam cat# ab18058) in 1% BSA, 0.1% Tween‐20 solution. Cells were counterstained with secondary antibody (Alexa Fluor 488, Thermo Fisher) and DAPI‐containing mounting medium (Vector Laboratories) and imaged on a Zeiss Axio Observer microscope and AxioVision software. A custom CellProfiler pipeline was used to identify vinculin intensity as well as vinculin area normalized for cell size. Fifteen cells pooled across three independent biological experiments were used to calculate vinculin content.

### 2.13 Gene expression

Cardiac NRVMs were grown on glass or 10 kPa substrates with fibronectin or fibronectin and TTR fibrils for 72 h. Total RNA was purified using Maxwell^®^ RSC simplyRNA Cells (Promega) with the inclusion of a DNAse treatment step. RNA samples were quantified using NanoDrop™ One Spectrophotometer (Thermo Scientific) and analyzed for integrity using Agilent 4200 TapeStation. Levels of remaining DNA were checked using a Qubit fluorometer (Invitrogen). Sequencing and gene expression statistical analysis were performed as previously described (Dittloff et al., 2021). Pathway analysis of differentially expressed genes was performed in Ingenuity Pathway Analysis. Visualization of differential expression and pathway analysis was performed via Integrated Differential Expression and Pathway analysis (Ge et al., 2018). Data are available in Figure S1–S3.

### 2.14 Statistical analysis

Data for all experiments were collected and organized using Microsoft Excel software (Microsoft) and histograms and statistical analysis were performed with GraphPad Prism software (GraphPad Software). Data are expressed as mean ± SEM for histograms or relative frequency distributions. Statistical significance was determined via calculation of two‐tailed student's *t*‐test, ordinary one‐way ANOVA, Fisher's exact test, or Mann–Whitney test, specified where appropriate.

## 3 RESULTS

**FIGURE 1:**
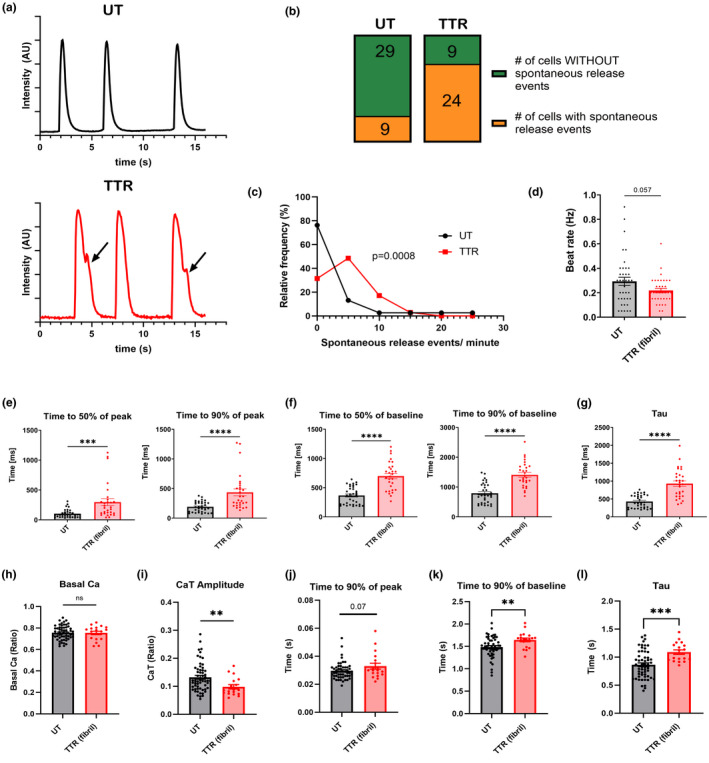
Cardiomyocytes cultured on transthyretin (TTR)‐coated surfaces have altered calcium‐handling properties and more spontaneous release events. (a) NRVMs grown on 10 kPa hydrogels and loaded with Fluo‐4, AM have increased spontaneous release events as indicated by black arrows when surfaces are coated with TTR fibrils. The number of NRVMs containing at least one spontaneous release event is significantly increased when 10 kPA surfaces are coated with TTR fibrils (b). An increased relative frequency of spontaneous release events per time (c), along with a decreased beat rate (d) is observed on TTR fibrils. Analysis of calcium transients indicates that NRVMs cultured on 10 kPa TTR fibril‐coated surfaces have slower rise time (e), prolonged duration (f), and an increased calcium decay transient *tau* (g). For all panels (a–g), *n* = 38 and 33 cells for UT and TTR, respectively. ***p < 0.001, ****p < 0.0001, ns = not significant. Wilcoxon test used for (c), Fisher's Exact Test for (d), and student's two‐tailed *t*‐test for (e–g), respectively. Consistent with the results from NRVMs, field stimulation (0.5 Hz) induced calcium transients in isolated mouse atrial myocytes exhibited an unaltered baseline calcium ratio (h), reduced calcium transient amplitude (i), prolonged duration (j, k) and increased *tau* (l). For (h–l), 59 individual cells were analyzed for UT and 18 individual cells were analyzed for TTR fibril conditions. ns = not significant, **p < 0.01, ***p < 0.001, (student's two‐tailed *t*‐test)

Several different approaches were used to assess how cardiac myocytes responded to TTR with respect to changes in their calcium‐handling properties (Figure 1). NRVM cultured on soft (10 kPa) hydrogel and loaded with Fluo‐4 had significant alterations in the amplitude and kinetic of their calcium transients when the TTR fibrils were present. Notably, NRVM cultured on TTR fibrils or on untreated surfaces exhibited calcium transients at regular frequency with those on TTR fibrils trending to lower frequencies (Figure 1d). However, NRVMs on TTR fibrils exhibited irregular spontaneous calcium release events with the random occurrence and of lower amplitude than the calcium transient (Figure 1a, denoted by black arrows). Overall, the number of cells presenting with at least one spontaneous release event (Figure 1b) and the frequency of events in individual cells was higher in NRVMs cultured on TTR fibrils (Figure 1c). Calcium transient rise time (Figure 1e), time to baseline (Figure 1f), and decay constant tau (Figure 1g) were all significantly increased when NRVMs were grown on TTR fibrils. Similar alterations were found in freshly isolated mouse atrial myocytes that were plated on laminin and TTR fibril‐coated glass coverslips for 2 h and loaded with Indo‐1. Field stimulation‐induced calcium transients (0.5 Hz) from atrial myocytes plated on TTR exhibited unaltered basal calcium ratio (Figure 1h), an attenuated calcium transient amplitude (Figure 1i), a prolonged calcium transient duration (Figure 1j,k), and decay constant (τ) (Figure 1l).

**FIGURE 2:**
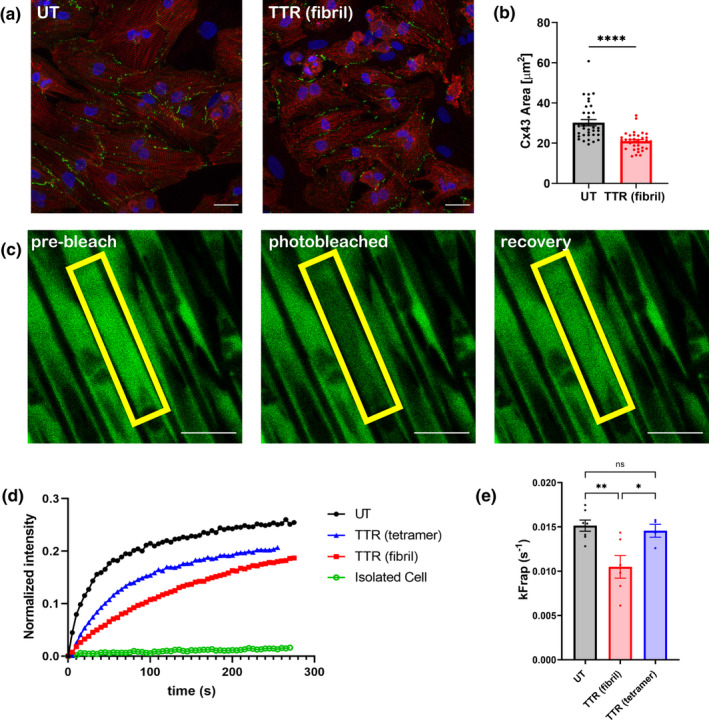
Cardiomyocytes cultured on TTR‐coated surfaces have decreased gap junction protein expression and altered intercellular electrical coupling. (a) Immunofluorescent images of NRVMs grown on glass substrates without (UT, left) or with (TTR fibril, right) TTR fibrils and stained for α‐actinin (red), connexin 43 (green), and DAPI (blue). Scale =25 μm. (b) Quantification of immunofluorescence indicates lower connexin 43 area normalized for cardiomyocytes in each field of view. *n* = 35 images per condition, ****p < 0.0001 (student's *t*‐test). (c) Example of Gap‐FRAP assay of calcein, AM‐loaded NRVMs grown in microgrooved surfaces. A yellow ROI around one aligned cell is photobleached and fluorescence intensity is allowed to recover, relative to the non‐bleached area. (d) Example FRAP curve of aligned, calcein, AM‐loaded cells. Importantly, single cells without neighboring cells show very slow recovery of fluorescent signal (green line). (e) FRAP constant, k, is decreased for NRVMs grown on TTR fibril‐containing microgrooves but not on TTR tetramer‐containing microgrooves. *n* = 4–7 experiments per condition, *p < 0.05, **p < 0.01 (One‐way ANOVA)

Significant structural and functional differences were found for gap junction connectivity for NRVMs when grown on both flat glass and microgrooved PDMS surfaces coated with TTR fibrils (Figure 2). Immunofluorescent detection of connexin 43 of NRVMs grown on glass was used to show the morphologic distribution covered by gap junctions (Figure 2a). Overall, cells on TTR fibrils have reduced gap junction protein expression, confirmed by quantification of the connexin 43 pixel area per cardiomyocyte (Figure 2b). Striations seen by α‐actinin staining on flat glass substrates with TTR fibrils appear qualitatively less well‐organized, but this was not quantified for cells grown on glass, see below for other substrata.

Functional assays for intercellular coupling were done on NRVMs loaded with gap junction‐permeable calcein‐AM and aligned by growing on the stiff, microgrooved PDMS substrates coated either with TTR fibrils or tetramers. Typical raw images before photobleached and after recovery are shown (Figure 2c). The time course for recovery of the dye from neighboring unbleached cells (normalized to intensity) is shown for untreated, with TTR fibril or TTR tetramers (Figure 2D). Note that an isolated cell without an adjacent neighbor (lowest trace) never recovers, confirming that gap junction coupling with a loaded neighbor is necessary. Interestingly, the TTR fibrils significantly disrupted the gap junction connectivity between cells, but the tetrameric TTR did not (Figure 2e).

**FIGURE 3:**
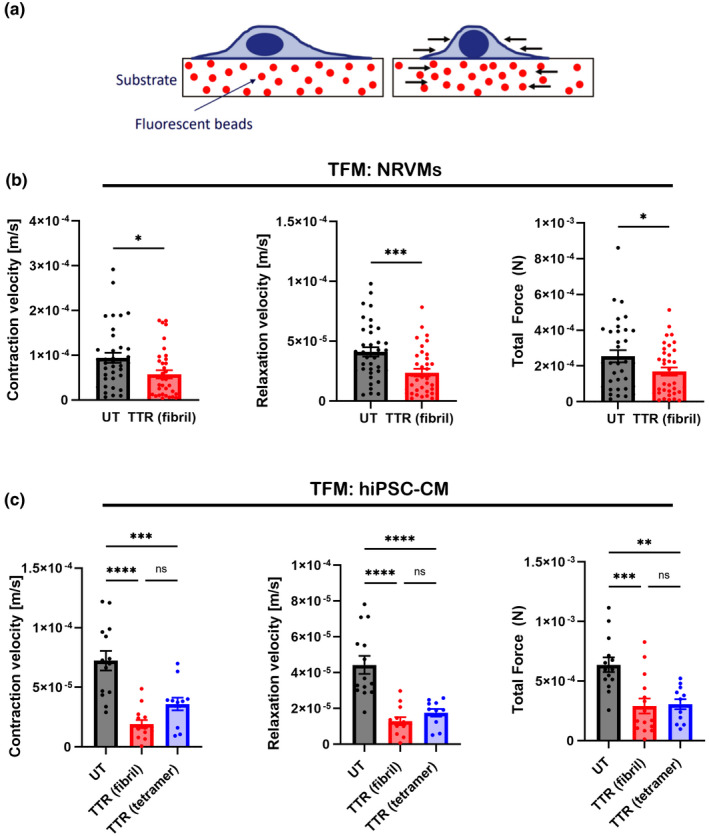
Cardiomyocytes cultured on TTR‐coated surfaces have decreased contractile profiles and force generation. (a) Overview of traction force microscopy technique in which displacement of fluorescent microbeads contained in deformable 10 kPa hydrogels is measured. (b) NRVMs on 10 kPa TTR fibril‐coated substrates have slower contraction and relaxation velocities, with a corresponding decrease in total force generation per contraction. *n* = 32–38 individual cells per condition, *p < 0.05, ***p < 0.001 (student's *t*‐test). (b) Similarly, hiPSC‐CMs on 10 kPa TTR fibril‐ or tetramer‐coated substrates have slower contraction and relaxation velocities, with a corresponding decrease in total force generation per contraction. *n* = 12–15 individual cells per condition, **p < 0.01, ***p < 0.001, ****p < 0.0001, ns = not significant (One‐way ANOVA)

Changes in contractility produced by NRVMs and human iPS‐CMs in the presence or absence of TTR were determined by traction force microscopy for cells grown on 10 kPa hydrogel containing fluorescent microbeads where the spontaneous beating displaces the beads in the substrate below (Figure 3a). In both cell types, there is a significant decrease in the contractile characteristics in the presence of TTR analyzed from video recordings. NRVMs with TTR fibrils on the substrate showed a reduced total force, contraction, and relaxation velocities (Figure 3b). These contractile differences were confirmed for hIPS‐CMs on TTR fibrils (Figure 3c). In addition, the TTR tetramer gave significantly lower force and velocity profiles to untreated hIPSC‐CMs, but these did not differ from the TTR fibril exposure (Figure 3c).

**FIGURE 4:**
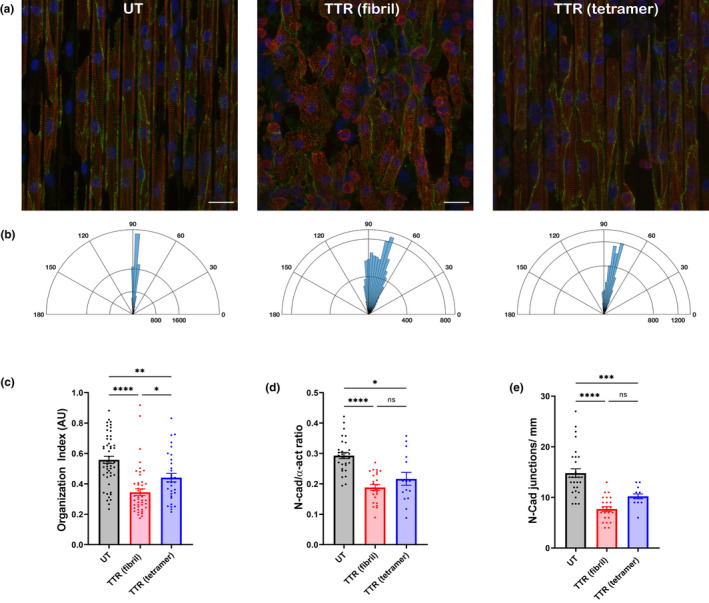
Cardiomyocytes cultured on TTR‐coated microgrooves have decreased sarcomere organization and decreased mechanical junction content. (a) Immunofluorescent images of NRVMs grown on microgrooved substrates stained for α‐actinin (red), N‐cadherin (green), and DAPI (blue). Scale =25 μm. (b) Corresponding rose plot of sarcomere angles relative to groove axis for each image shown above; 90° indicates perfect longitudinal alignment. Observe a wider distribution of sarcomere angles on TTR fibril‐containing (middle) and TTR tetramer‐containing (right) grooved substrates. (c) Relative to NRVMs grown on untreated microgrooved surfaces, sarcomeres are highly disorganized on surfaces containing TTR fibrils, but to a lesser degree on surfaces containing TTR tetramers. *n* = 35 images per condition, *p < 0.05, **p < 0.01, ****p < 0.0001 (One‐way ANOVA). (d) N‐cadherin content, normalized to α‐actinin content, is decreased on both TTR‐containing substrates; (e) Formed N‐cadherin positive cell‐cell junctions are decreased on both TTR‐containing substrates. *n* = 15–30 images per condition, *p < 0.05, **p < 0.01, ***p < 0.001, ****p < 0.0001, ns = not significant (One‐way ANOVA)

In addition to the gap junction and its implied electrical connectivity of cardiomyocytes, the mechanical connectivity and sarcomeric cytoskeletal organization were quantified for NRVMs grown on stiff PDMS microgrooves with deposited tetrameric or fibrillar TTR (Figure 4). Observation of the immunofluorescent images stained for α‐actinin reveals much greater sarcomeric disorganization in orientation and amount with TTR fibrils, present but less obvious with the tetramer (Figure 4a). The polar rose plots display this orientation visually (Figure 4b), with further quantification of the organization index defined in the methods shown (Figure 4c). Clearly, the presence of TTR fibrils is a significant disruptor of the ability of an NRVM to align within the microgrooves compared to untreated surfaces, and the tetramer disorganizes the sarcomeric striation pattern but less so.

The immunofluorescent staining for N‐cadherin was used as an indicator of the mechanical connectivity between NRVMs and shows the normal side‐to‐side and end‐to‐end distribution for untreated cells (Figure 4a). The notable changes in both the amount and location were greatest when TTR fibrils were deposited on the microgrooves and also obvious with the tetramer (Figure 4a). Indeed, the N‐cadherin content normalized to α‐actinin was significantly reduced for both TTR‐containing substrates (Figure 4d). Additionally, fewer junctions at the myocyte ends were detected for aligned NRVMs grown on microgrooved substrata with either of the deposited TTR species, as measured by N‐cadherin positive junctions per millimeter parallel to the groove axis (Figure 4d), indicating altered mechanical connectivity due to TTR deposition.

**FIGURE 5:**
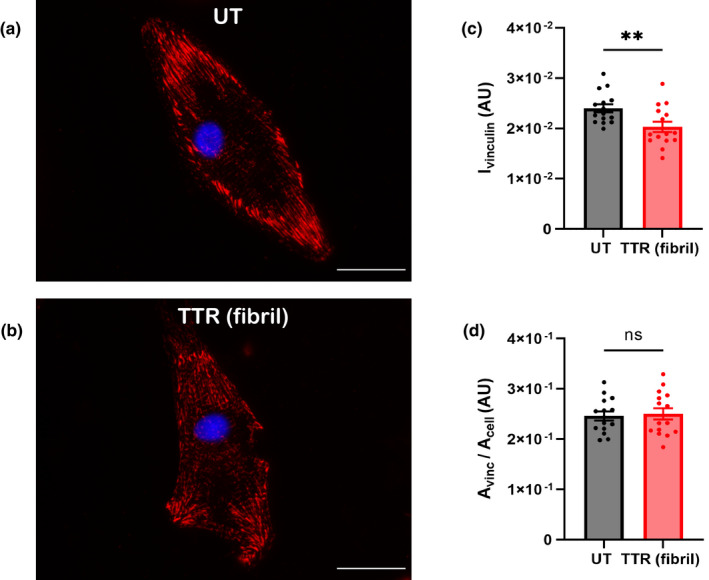
Cardiomyocytes cultured on TTR‐coated glass substrates exhibit decreased vinculin intensity. Immunofluorescent images of NRVMs grown on untreated (a) or TTR‐coated glass substrates (b) stained for vinculin (red) and DAPI (blue). Scale =25 μm. (c) Vinculin intensity is decreased for NRVMs grown on TTR‐containing substrates. (d) Focal adhesion area, relative to cell area, is unchanged. *n* = 15 cells per condition, **p < 0.01, ns = not significant (student's two‐tailed *t*‐test)

Another cell junction protein examined was vinculin, a component of the intercalated disc and the fascia adherens, known as the costamere in cardiac muscle. Here too, the immunofluorescent staining for vinculin differs in the presence of TTR fibrils for isolated NRVMs grown on glass. Note, a similar pattern for distribution but lower intensity staining in the cell periphery for NRVMs on TTR fibrils (Figure 5a,b). Quantification confirms the relative intensity of vinculin is significantly different (Figure 5c), but the actual area of the focal adhesion per cell was unchanged (Figure 5d).

**FIGURE 6:**
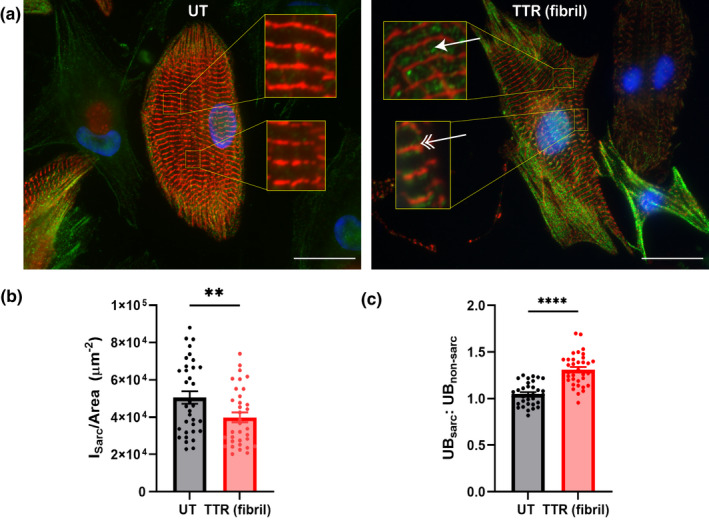
Cardiomyocytes cultured on TTR‐coated glass substrates have decreased sarcomere content and increased ubiquitination at the Z‐disc. (a) Immunofluorescent images of NRVMs grown on TTR‐coated glass substrates stained for α‐actinin (red), linkage‐specific (K48) ubiquitin (green), and DAPI (blue). Scale =25 μm. (b) Sarcomere intensity, normalized to the cell area, is decreased for NRVMs grown on TTR‐containing substrates. (c) Intensity ratio of ubiquitin (K48 linkage) at the sarcomere to non‐sarcomeric ubiquitin is increased for NRVMs grown on TTR fibrils. *n* = 35 cells per condition, **p < 0.01, ****p < 0.0001, (student's two‐tailed *t*‐test)

Given all the major changes seen in the organization of the sarcomeres, along with the gap and focal adhesion junctions, it was important how other cellular processes might be changed. Loss of cellular protein is accompanied by polyubiquitination, assessed by an antibody for K48 linkage. Immunofluorescent images of α‐actinin (Figure 6a and prior images Figures 2 and 4) strongly suggest a reduction in total sarcomeric content. Confirmation that the sarcomere content per cell was reduced in NRVMs on TTR fibril‐coated surfaces was confirmed quantitatively (Figure 6b). The localization of ubiquitin in untreated cells is mostly near the cell periphery and not sarcomeric, but with TTR, the ubiquitin also appears to be sarcomeric, mainly in the I‐band (Figure 6a). To limit the assessment to the sarcomere, measurement of the ubiquitin was restricted to that co‐localized with α‐actinin pixels and their surrounding 0.65‐μm annulus and found to be significantly higher with TTR fibrils (Figure 6c). The ubiquitin appears to be in transverse line at different locations in the I‐band adjacent to the Z‐disc.

## 4 DISCUSSION

This report details the effects of TTR tetramers and fibrils on the structure and function of various cardiomyocytes (neonatal rat ventricle, derived from human iPSC, or adult mouse atrium) when cultured on various substrates. NRVMs have more spontaneous calcium release events, and both NRVMs and adult mouse atrial cardiomyocytes have slower calcium transients. Moreover, NRVMs have decreased gap junction protein expression and attenuated intercellular coupling. Furthermore, the normal myofibrillar architecture is disorganized, with reduced sarcomeric and mechanical junction content. Importantly, taken together, these changes lead to decreased contractile rise and fall times and reduced force generation as measured by traction force microscopy. Subcellular changes in response to TTR are accompanied by increased ubiquitin localization in the sarcomere. The abnormal mechanical function seen in vitro may play an underlying role in the maladaptive diseases that occur when amyloidogenic TTR is deposited in the aging human heart.

Since there is no whole animal system producing TTR amyloid in the heart, it was necessary to use a model system to examine cardiomyocyte responses. Here, a variety of approaches were used with different cardiomyocyte preparations to encompass the range of parameters known to be a problem in the diseased heart. The primary approach was to deposit tetrameric or fibrillar TTR on substrates as done in a study on fibroblast structure and function (Dittloff et al., 2021). TTR fibrils are formed in a dynamic process that involves the exchange of unfolded oligomers with actively forming fibrils (Groenning et al., 2015) noting that there will always be some contribution from monomers and tetramers in vivo, and in this culture system in vitro.

The deposited TTR system is used again here with the addition of using the normal myocardium stiffness of 10 kPa so that the cells could shorten (Engler et al., 2008). A softer substrate avoids any dysfunctional changes due to higher stiffness (Chang et al., 2021). Stiffness is a major trigger of several mechanosignaling pathways making it a key factor in the regulation of muscle cell size (Russell & Solís, 2021; Solís & Russell, 2019). In some experiments, myocytes from NRVMs and iPSCs were grown in microgrooves so that the effects of TTR could be examined in aligned myocytes with correct electrical and mechanical junction distribution (Motlagh, Hartman, et al., 2003). Not all parameters can be examined in each substrate, for example, soft surfaces do not retain their microtexture, and stiff substrata do not permit cell shortening. Therefore, selective use of various myocyte types and appropriate substrata were made to recapitulate the parameter being studied to assess the effects of TTR.

Heart failure is characterized by decreased ability of the heart muscle to pump enough blood to meet the body's needs for nutrients and oxygen. Abnormality in the structure and function of the sarcomere for effective force production is, therefore, of serious clinical concern. In humans, it takes decades for heart failure to develop, but here, the cultured cells show effects within hours (adult mouse atrial) or days (NRVMs and iPSC‐CMs). This may reveal a limit to the studies; herein, the whole heart tissue is not used, or the “dose” of the TTR may be excessive. Nonetheless, the changes in the isolated cells may be indicative on the effects of TTR.

In this study, cardiomyocytes (NRVM, adult atrial) plated in the presence of TTR fibrils exhibited aberrant calcium transient kinetics. In both cell types, the calcium transient duration and decay constant were significantly prolonged consistent with an attenuated activity of the sarco‐/endo‐plasmic reticulum ATPase (SERCA) that accounts for ~80% of calcium removal at the end of a transient. A shift in calcium removal from SERCA to the sodium‐calcium exchanger would also be consistent with the prolonged action potential duration previously described. While changes in SERCA expression in either cell type cannot be ruled out, the short time frame (2h) in which fibrils induced these changes in adult myocytes perhaps suggests post‐translational protein modifications. Attenuated SERCA activity has been reported as a consequence of increased cellular ROS production. This would be consistent with TTR‐induced changes previously reported and therefore not repeated here (Sartiani et al., 2016).

Calcium transients in NRVMs further exhibit an attenuated rise time and increased number of spontaneous calcium release events. The latter represents a mechanism of triggered activity (Campos et al., 2015) that has been linked to the onset of atrial fibrillation which is observed in up to 70% of wtATTR patients and provides clinical challenges (Mints et al., 2018). Manifestation of both atrial and ventricular arrhythmias is prevalent in wtATTR. Ventricular arrhythmia is a prognosticator of sudden cardiac death in cardiac amyloidosis patients, but clinical management and use of implantable cardioverter defibrillators in these patients are not fully understood (Khanna et al., 2020).

The current data cannot distinguish whether the underlying mechanism of the spontaneous release events was caused by early after depolarization due to a prolonged action potential duration or caused by spontaneous release events due to increased ryanodine receptor (RYR) open probability. The fact that the basal calcium is not increased and that the calcium transient amplitude is attenuated does not support sarcoplasmic reticulum calcium overload as a mechanism of increased RYR activity. A possible speculation is that a prolonged duration of a calcium transient‐ and/or action potential triggers calcium release from the RYR, whose open probability might be shifted to lower calcium concentrations by increased cellular ROS production (Sartiani et al., 2016). Further experiments using calcium channel blockers and electrophysiological techniques could explore calcium transient and calcium‐handling mechanisms in TTR amyloidosis.

In cardiac tissue, the expression of connexin 43, the predominant gap junction protein in atrial and ventricular myocytes, is critical to maintain a low intercellular resistance and continuous action potential propagation. Remodeling of gap junction complexes is associated with a heterogeneity in excitation spread and is associated with the development of arrhythmia (Severs et al., 2004). In this study, the culture of NRVMs on TTR fibrils reduced the number of connexin 43 positive gap junction clusters and attenuated functional intercellular coupling as demonstrated by the delayed FRAP dynamics. It is not known whether the change in functional intercellular coupling is regulated by the connexin 43 expression level or by changes in subcellular localization, but both could affect the isotropy of conduction, thereby facilitating the occurrence of reentrant excitation.

The presence of a highly organized and dense sarcomere structure is of utmost importance for force generation and correct heart functioning (Solís & Solaro, 2021). There is a significant decrease in myocyte alignment and disarray in pathological conditions, which can be linked with ventricular arrhythmias and sudden cardiac death (Ariga et al., 2019). Here, the presence of TTR resulted in decreased sarcomeric content, disorganization of the striations, and cell alignment, which could corroborate these clinical findings. Indeed, reduction in force and slower contractile parameters were confirmed by the traction force microscopy analysis in NRVMs and human iPSC‐CMs plated on soft 10 kPa TTR‐coated substrates.

Mechanical force is transferred from the end of one cardiomyocyte to another through the fascia adherens junctions (intercalated discs), and laterally to the ECM through costameres (Russell et al., 2010; Solís & Solaro, 2021; Solís & Russell, 2021). These mechanical junctions are essential for normal cardiac function (Pruna & Ehler, 2020). Both vinculin and N‐cadherin are membrane‐bound proteins that strongly anchor two neighboring cells were significantly reduced by TTR presence. Knock‐out of N‐cadherin in transgenic mice had dissolution of the intercalated disc that led to sudden death (Kostetskii et al., 2005). Mechanical junctions are disrupted in the failing heart which is accompanied by faulty mechanosignaling pathways (Samarel et al., 2013; Sit et al., 2019). The reduction of the mechanical junctions by TTR is thus another index of the pathological pathways that TTR may be triggering.

Cardiac amyloidosis is accompanied by fibrosis and wall stiffness, which may be partially explained by the finding that cardiac fibroblasts cultured on deposited TTR fibrils had increased proliferation and migration rates (Dittloff et al., 2021). Additionally, patients with TTR amyloidosis have increased inflammatory profiles that correlate with disease progression (Azevedo et al., 2019; Hein, Knoll, et al., 2021), which was also seen in fibroblast culture (Dittloff et al., 2021). Note that the primary myocyte cultures used here also contain fibroblasts, so upregulation of the inflammatory genes was to be expected (see Figures S1–S3 for pathway analysis of RNA‐seq data). Inflammation can accompany various cardiomyopathies and myocarditis (Asatryan et al., 2021), demonstrating the importance of activating an inflammatory response, perhaps directly by the exogenous TTR molecules.

Results in this paper show significant loss of sarcomeres after only a few days of exposure to TTR with an increased amount of ubiquitin seen in the sarcomeres. During the loss of sarcomere content seen with TTR, one must expect filament disassembly generating an excess of unassembled proteins, which are known in other situations to become tagged for degradation and reprocessing (Khalil & Xiao, 2018). For example, myocardial tissue from patients with dilated cardiomyopathy has increased levels of myofibrillar ubiquitination associated with dysfunctional protein degradation through autophagy via the co‐chaperone BAG3 (Martin & Kirk, 2020; Martin et al., 2021). Loading stabilizes the sarcomeric structure and decreases the rate at which proteins are exchanged with the cytoplasm (Russell & Solís, 2021; Solís & Russell, 2019). A stable sarcomere protects proteins within the filamentous lattice and explains the long half‐life before the degradation. However, once free in the cytoplasm, myosin and actin degradation increases as sarcomeres disassemble (Byron et al., 1996) and the half‐life of proteins is greatly reduced in both cardiac and skeletal muscles (Samarel et al., 1992) due to ubiquitin tagging activation of the ubiquitin proteasome pathway (Wang et al., 2020). Increased ubiquitination of sarcomeric protein is found on immobilized rat skeletal muscle (Ryder et al., 2015). Dysregulation of the ubiquitin signaling pathway also results in atrophy and sarcomere disorganization (Perera et al., 2011). Thus, TTR presence triggers sarcomere disassembly and the ubiquitin‐proteasome pathways in this system.

## 5 SUMMARY

Cardiac myocytes perform the mechanical work to pump blood through the body. Therefore, any pathology that compromises myocardial function may jeopardize the maintenance of cardiovascular circulation. This work identifies how deposited TTR dysregulates sarcomere structure and function using cardiac myocytes derived from human iPSC or isolated from neonatal rat ventricle and adult mouse atrium. Myocytes exposed to TTR had decreased contraction and relaxation profiles, with altered calcium kinetics and prolonged transients. TTR deposition also caused decreased gap junction content and intercellular electrical coupling in myocytes, along with disorganized sarcomere structure and increased ubiquitin localization to the sarcomere. These discoveries may lead to further understanding of the electromechanical coupling seen in heart failure due to wtATTR and may guide future research into therapeutic approaches for this cardiac disease in the elderly.

## CONFLICT OF INTEREST

No conflicts of interest, financial or otherwise, are declared by the authors.

## AUTHOR CONTRIBUTIONS

K.T.D. and B.R. conceived and designed research; K.T.D., E.S., C.S., and K.B. performed experiments; K.T.D., E.S., C.S., K.B., and B.R. analyzed data and interpreted results of experiments; K.T.D. and E.S. prepared figures; K.T.D., E.S., and B.R. drafted the manuscript; K.T.D., E.S., C.S., K.B., and B.R. edited and revised manuscript; K.T.D., E.S., C.S., K.B., and B.R. approved the final version of the manuscript.

## Supporting information



Figure S1–S4
