# Supplementary material for: Transthyretin deposition alters cardiomyocyte sarcomeric architecture, calcium transients, and contractile force
Source: Physiol Rep. 2022 Mar 9;10(5):e15207. doi: 10.14814/phy2.15207 (PMC8906053; doi:10.14814/phy2.15207)
Supplement: Supplementary file 1 — Figure S1–S4 [file PHY2-10-e15207-s001.docx]

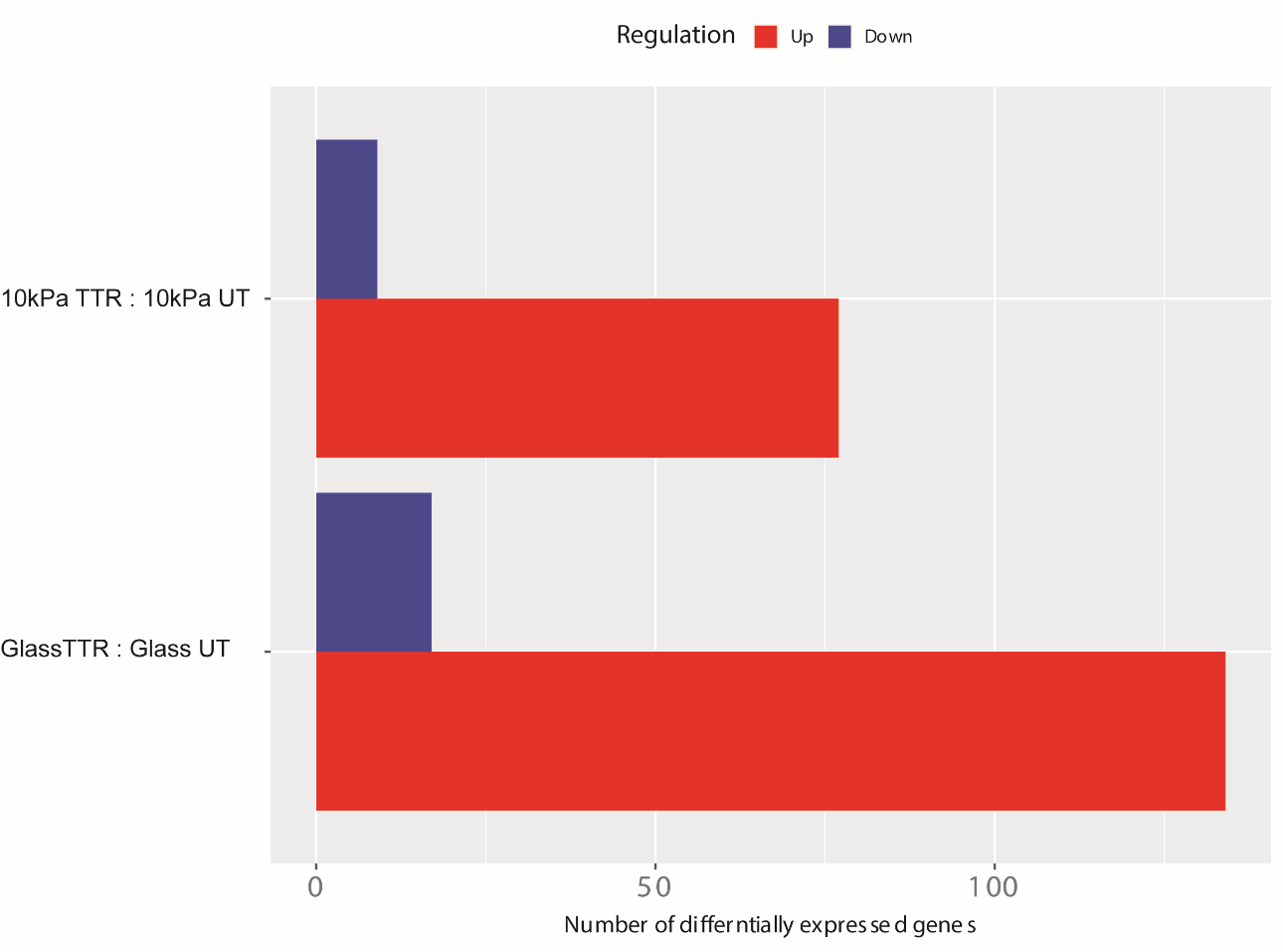


**Fig S1.** Differentially expressed genes determined by DESeq2 method. Cutoff for false discovery rate (FDR) was 0.1, with a minimum fold change cutoff of 1.5.


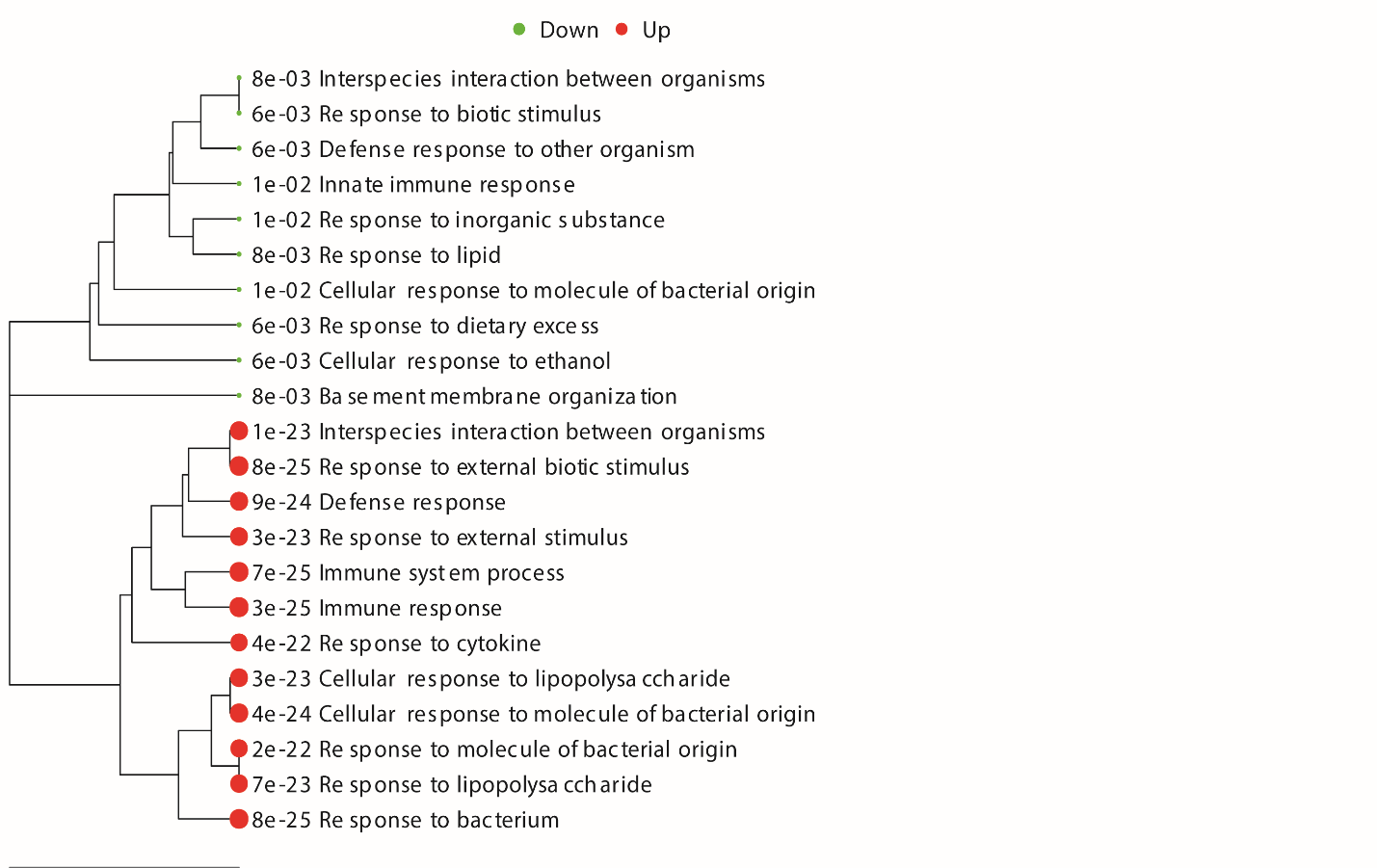


**Figure S2.** Enrichment plot of most significantly changed Gene Ontology: Biological Process (GO:BP) pathways identified by RNA-seq for NRVMs grown on glass substrates.


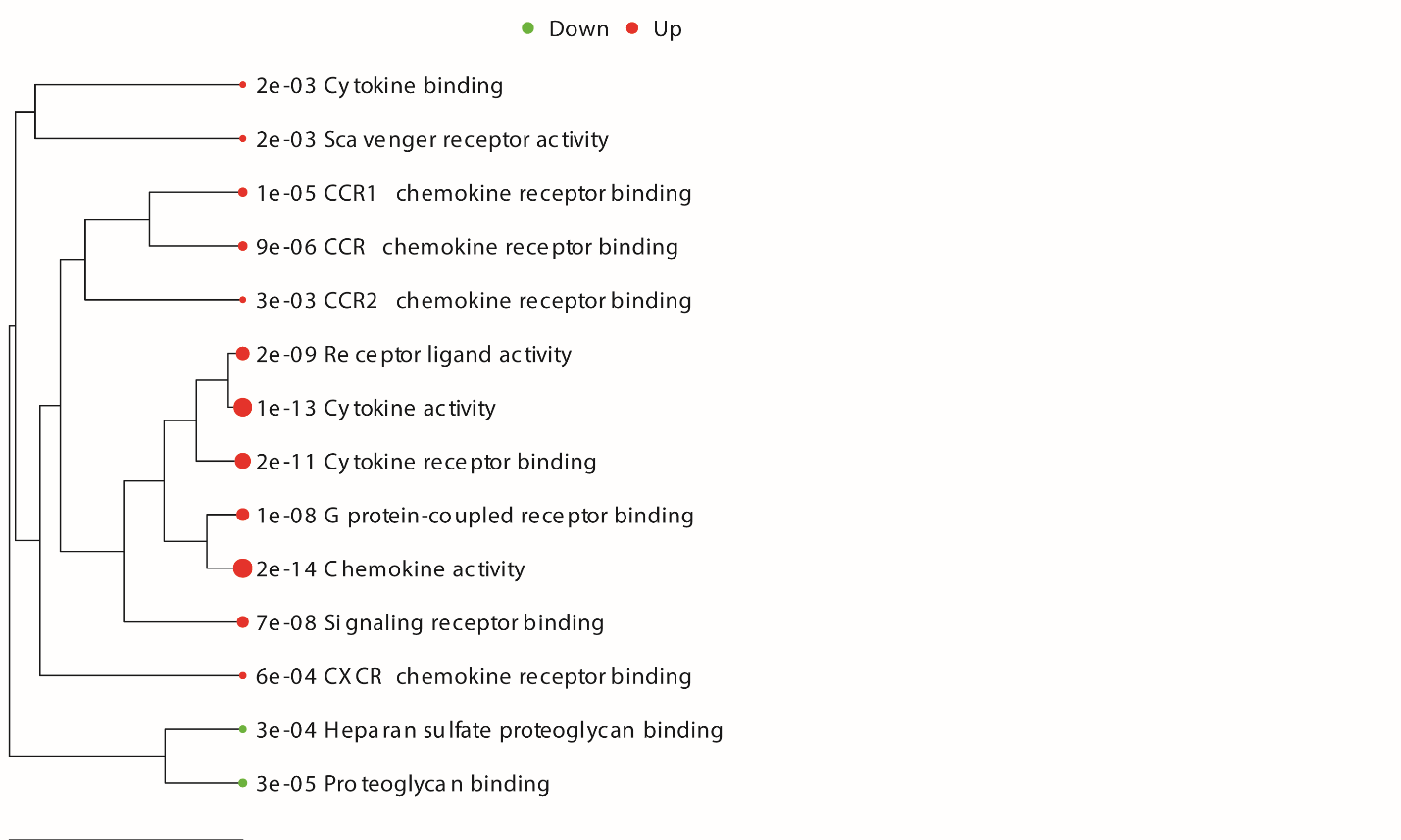


**Figure S3.** Enrichment plot of most significantly changed Gene Ontology: Molecular Function (GO:MF) pathways identified by RNA-seq for NRVMs grown on glass substrates.


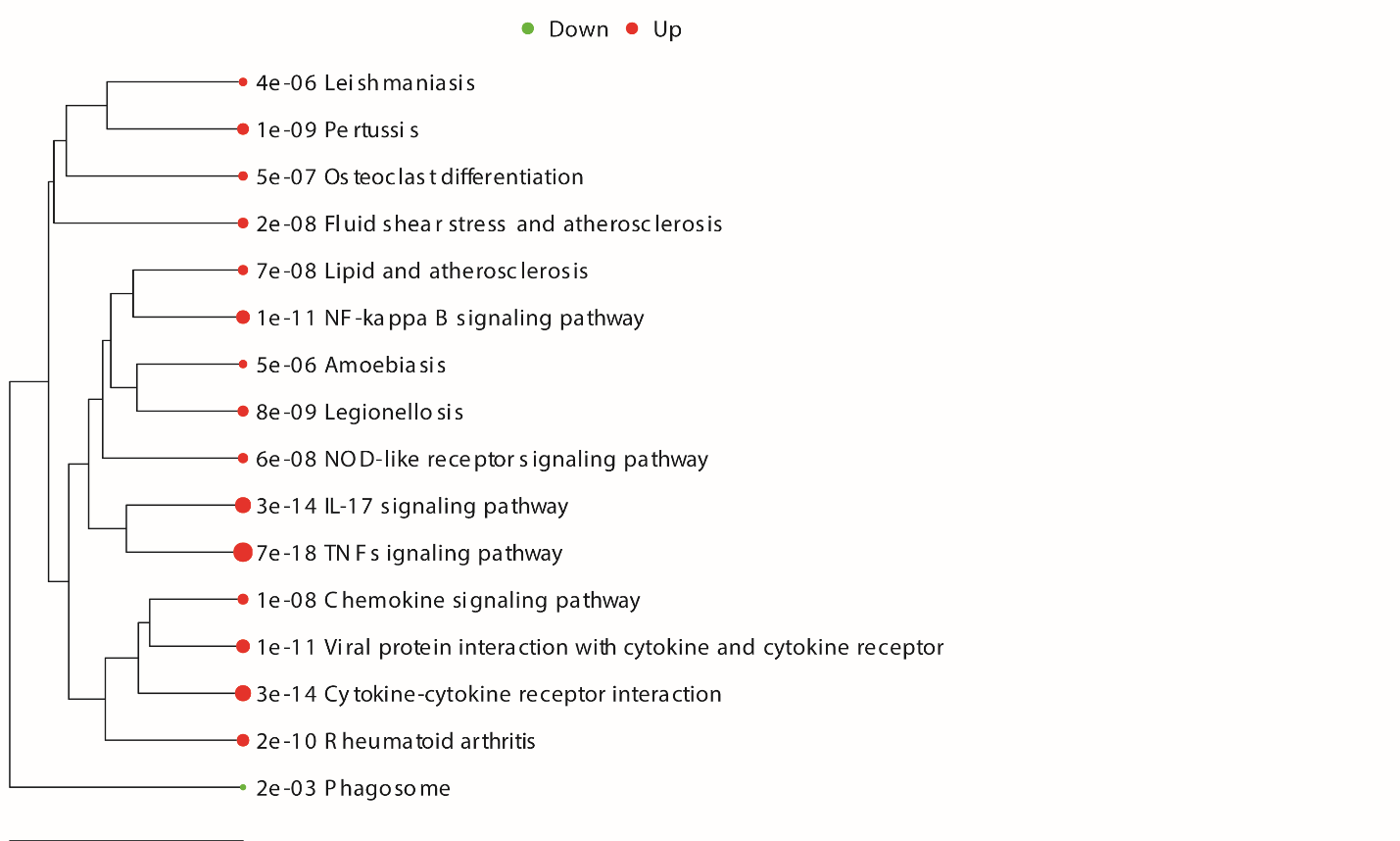


**Figure S4.** Enrichment plot of most significantly changed KEGG terms identified by RNA-seq . for NRVMs grown on glass substrates
